# Perceived stress precedes declines in Well-being: A prospective study of stress, well-being, hair cortisol, and low-grade inflammation in hospital employees

**DOI:** 10.1016/j.bbih.2025.101158

**Published:** 2025-12-11

**Authors:** Monica T. Jones, Rachael A. Cronin, Mathew D. Marques, Matthias Weigl, Nicolas Rohleder, Linda Becker, Helena C. Kaltenegger, Bradley J. Wright

**Affiliations:** a School of Psychology and Public Health, La Trobe University, Wodonga, Victoria, 3689, Australia; b School of Psychology and Public Health, La Trobe University, Melbourne, Victoria, 3086, Australia; c Institute for Patient Safety, University Hospital Bonn, Bonn, Germany; d Institute and Clinic for Occupational, Social and Environmental Medicine, University Hospital, LMU Munich, Munich, Germany; e Health Psychology, Department of Psychology, Friedrich-Alexander-Universität Erlangen-Nürnberg, Erlangen, Germany

**Keywords:** Positive affect, Chronic stress, C-Reactive protein, HPA axis, RI-CLPM

## Abstract

**Objective:**

Chronic low-grade inflammation may help explain the relationship between stress, well-being, and disease, but the pathway and temporal order have not yet been tested prospectively. To understand the pathways between perceived stress, well-being, C-reactive protein, and hair cortisol, we investigated the temporal ordering of these variables in a sample of hospital employees.

**Methods:**

Random-intercepts cross-lagged panel models were conducted using three 6-monthly waves of data collected from new employees at a German hospital (*N* = 296, 77.7 % female, M age = 28.59) in a prospective cohort study. Self-reported data on perceived stress and well-being, hair strands for hair cortisol concentration, and capillary blood samples for C-reactive protein were collected for analysis.

**Results:**

While our study did not support a causal relationship between changes in stress levels and later changes in either hair cortisol or low-grade inflammation, we provide evidence to suggest that increases in perceived stress led to later decreases in well-being. In contrast, changes in well-being did not predict changes in perceived stress levels.

**Conclusion:**

This is the first prospective repeated-measure study to examine the temporal associations between stress, well-being, hair cortisol concentrations, and chronic low-grade inflammation. Our analyses suggest that perceived stress in this sample precedes changes in well-being, highlighting the importance of prevention and early intervention.

## Highlights

• Stress increases predicted lower well-being 6 months later, not vice versa.
• No links found between stress or well-being with HCC or CRP over time.
• Stress, HCC, CRP stable; well-being declined for nearly half of participants.
• Early stress reduction may prevent reduced well-being.

## 1 Introduction

The pathway and temporal order linking psychosocial stress and well-being to dysregulated physiological processes and the development of disease states are not yet understood. Higher perceptions of psychosocial stress have been associated with anxiety, depressive symptoms (Wiegner et al., 2015), and cardiovascular disease (Musey et al., 2020; Nabi et al., 2013). While much of the literature has focused on the relationship between negative psychological states and ill-health, well-being should be considered as health is a person's overall physical, mental and social state, not just the absence of illness (World Health Organization, 2020). In terms of physiological correlates, emerging evidence has demonstrated that low-grade inflammation, as defined by many, and used in the present study as C-reactive protein (CRP) values between 3 and 10 mg/L, may play an important role in the transmission of poor psychosocial stress and well-being to disease states (Piantella et al., 2021a). That said, while the relationship between perceived stress, well-being, and physiological indicators of stress and ill-health has been demonstrated, the temporal order of this relationship remains unclear. Clarifying whether psychosocial factors, such as perceived stress or well-being, precede or follow physiological responses, including dysregulated cortisol production and low-grade inflammation, is essential for informing early intervention and prevention strategies.

As our focus is on working adults, we will concentrate our review of the psychosocial stress literature on studies that assess perceived stress (i.e., feelings of stress) using the Perceived Stress Scale (PSS4; Cohen et al., 1983) or a measure of workplace stress. Perceived stress has been linked to systemic inflammation, often inferred from moderate elevations in biomarkers of acute-phase proteins (e.g., CRP) or inflammatory cytokines (e.g., interleukin-6 [IL-6]) in blood or saliva (Rohleder, 2019). Acute inflammation in the short term is an adaptive response; however, chronic low-grade inflammation may lead to the development of chronic disease (Cifuentes et al., 2025; Couzin-Frankel, 2010; León-Pedroza et al., 2015). Although research directly linking perceived stress to low-grade inflammation remains limited, literature reviews indicate that workplace stress is often positively associated with inflammatory markers, though the strength and consistency of this relationship vary across studies (Kaltenegger et al., 2021; Siegrist and Li, 2017; Viljoen and Thomas, 2021).

The association between psychosocial stress and low-grade inflammation across cross-sectional studies is mixed, with some reporting only weak positive associations between workplace stress and inflammation (Eguchi et al., 2023), while others suggest stronger associations in men than in women (Siegrist and Li, 2017). Longitudinal research has shown that higher levels of perceived stress were associated with elevated CRP levels in women but not in men (Barbosa-Leiker et al., 2014). Similarly, another found no prospective association between stress and CRP in an all-male sample (Wright et al., 2011). Similar to studies of perceived stress, workplace stress has also been inconsistently linked to inflammation. Eguchi et al. (2016) reported elevated CRP in women experiencing low supervisor support, whereas Shirom et al. (2008) found no association between job characteristics and CRP. Combinatorial measures of low-grade inflammation (IL-6, CRP) have also shown that workplace stress can increase low-grade inflammation (Duchaine et al., 2021). However, the absence of baseline physiological data limits the understanding of how psychobiology changed over time.

Emerging evidence suggests that lower well-being may also be linked to heightened low-grade inflammation. In a large-scale study (*n* = 16,952), low-grade inflammation was positively associated with depressive symptoms and negatively associated with well-being (Gialluisi et al., 2020). Similarly, elevated levels of IL-6 and CRP were related to increased depressive symptoms and reduced well-being, even after accounting for demographic and health-related variables (Millar et al., 2024). Longitudinal findings also support this link; in older adults, CRP was negatively associated with self-realisation (an indicator of well-being) but not with positive affect or life satisfaction (Fancourt and Steptoe, 2020). Together, these findings suggest reduced well-being may contribute to low-grade inflammation.

Given its influence on inflammation, cortisol, as a measure of HPA (hypothalamic-pituitary-adrenal) axis activity, has been widely studied. In the short term, cortisol promotes the release of pro-inflammatory cytokines and acute-phase proteins, while chronic exposure to high cortisol levels can lead to immune suppression and dysregulation (Alotiby, 2024). Measuring cortisol reliably has its challenges. As traditional methods of sampling are affected by pulsatile bursts, diurnal variations, and protocol compliance (see Stalder et al., 2017 for a comprehensive description), researchers have moved to measuring hair cortisol concentration (HCC). This method provides a retrospective and reliable measure of cumulative cortisol secretion over periods and is less sensitive to circadian rhythms and acute fluctuations (Stalder et al., 2017).

Investigations of the relationship between psychological stress and HCC have produced inconclusive findings. In a review of eleven studies examining the HCC-stress relationship, two found a positive relationship, two found a negative relationship, and the remaining seven found no significant relationship (Staufenbiel et al., 2013). Studies focusing explicitly on workplace stress have also yielded mixed results, with higher HCC linked to high work stress conditions in some samples (Qi et al., 2014; van der Meij et al., 2018), but others found no association with work stress(Gidlow et al., 2016; Herr et al., 2018; Janssens et al., 2017; Qi et al., 2014; van der Meij et al., 2018). The available research on workplace stress and HCC is further limited by a scarcity of prospective studies (Schaafsma et al., 2021). Penz and colleagues (2019) observed that increases in workplace stress at one year were related to a reduction in HCC two years later, suggesting blunted cortisol secretion. However, Mayer and colleagues (2018) found no prospective association between stressor demands or stress perceptions and HCC. In a healthy all-female sample, a positive association was found between well-being and HCC in older participants but not in younger ones (Smyth et al., 2016). In another study, no longitudinal association was found between HCC and well-being (Lawes and Eid, 2025).

Research examining prospective associations between psychological stress, well-being, and physiological indicators of stress, which also identifies the temporal pathways, is needed to better describe how perceived stress relates to psychobiological change. The present study will employ a series of secondary analyses (see Kaltenegger et al., 2024), with differing measures, timepoint comparisons, and questions, to investigate prospective associations between self-reported measures of perceived stress and well-being and biological measures of the HPA axis. Specifically, we aimed to 1) assess the reliability of the measures across time and determine if significantly meaningful changes (as measured by a Reliable Change Index) occurred over time; and 2) assess the direction, magnitude, and temporal ordering of the proposed perceived stress-well-being-low-grade inflammation-HCC pathway.

## 2 Materials and methods

### 2.1 Design

This prospective cohort study was conducted at a large university hospital in South Germany across three time points approximately six months apart. Data collection took place from June 2021 to November 2022, with the first measurement (Time 1) from 06 to 11/2021, the first follow-up measurement (Time 2) from 11/2021-05/2022, and the second follow-up measurement (Time 3) from 06 to 11/2022. Ethics approval was granted (20-0914), and the study was conducted under the ethical standards of the Declaration of Helsinki. The study protocol was registered *with the online registry OSF* (https://osf.io/9g4zn/overview). All participants provided written informed consent.

### 2.2 Participants and procedure

A total of *N* = 301 participants were included at the first measurement (Time 1); *n* = 241 were included at the first follow-up six months later (Time 2); and *n* = 200 participated at the second follow-up twelve months later (Time 3). Participants were recruited from new hospital employees after their obligatory pre-employment medical examinations. For follow-up measurements, the study team contacted participants and arranged individual appointments with the study team. In the final sample (*n* = 296), most participants were female (*n* = 230, 77.7 %), aged 16–60 years (*M* = 28.59, *SD* = 8.83), and the mean BMI was 23.74 (*SD* = 8.83). The majority of staff were carers (*n* = 84, 28.4 %), followed by medical care (*n* = 62, 20.9 %), research personnel (*n* = 41, 13.9 %), medical-technical personnel (*n* = 40, 13.5 %), administrative personnel (*n* = 16, 5.4 %), and other (such as midwives, therapists etc; *n* = 50, 16.9 %).

Statistical power analysis was not suitable for determining sample size, as this study does not involve hypothesis testing or significance testing. The practical constraints of conducting secondary analysis of earlier research meant the sample size was determined by Kaltenegger et al. (2024). A posteriori, participants who identified as non-binary-sex (*n* = 1) or participants who only provided biosamples without demographic or self-report data for comparison (*n* = 4) were excluded. The final sample size was *n* = 296.

### 2.3 Measures

Standardised self-report questionnaires and biomarker measurements were used. All key variables were measured at each of the three timepoints. Socio-demographic information for Time 1, including sex, age, and body mass index (BMI), along with employment details, is reported here (see section 2.2).

#### 2.3.1 Self-report measures

##### 2.3.1.1 Perceived stress

The Perceived Stress Scale 4 (PSS4; Cohen et al., 1983) is a well-established and global measure of psychological stress that captures a person's evaluation of their stress in the preceding month. The PSS4 comprises four items, such as “*In the last month, how often have you felt that you were unable to control the important things in your life?”*. Response options range from 0 = *never* to 4 = *very often.* Scores are summed, with higher scores indicating greater perceived stress. The PSS4 is a reliable and valid brief tool with adequate internal consistency (Karam et al., 2012; Sanabria-Mazo et al., 2024). The PSS4 demonstrated moderate to high internal consistency across occasions (Cronbach α = .641 (Time 1), α = .723 (Time 2), α = .760 (Time 3)). An intraclass correlation coefficient (ICC(A,3) of .754, 95 % CI [.701, .799]) was observed for PSS4, indicating good stability of PSS4 across the three timepoints.

##### 2.3.1.2 Mental well-being

Current well-being was assessed using the World Health Organisation – Five Well-Being Index (WHO-5; World Health Organization, 1998). The WHO-5 includes five positively phrased items regarding how respondents felt in the last two weeks (e.g., “*I have felt calm and relaxed”*) with response options ranging from 0 = *at no time* to 5 = *all of the time*. The raw score, ranging from 0 to 25, is calculated by totalling the responses and a percentage score, ranging from 0 to 100, is obtained by multiplying the raw score by 4. This provides a meaningful total score where 0 represents the absence of well-being, and 100 represents the highest possible well-being. The WHO-5 threshold for a clinically relevant change is 10 points (Topp et al., 2015). The reliability and validity of the WHO-5 are widely accepted (Topp et al., 2015). The WHO-5 demonstrated high internal consistency across occasions (Cronbach α = .866 (Time 1), α = .809 (Time 2), α = .817 (Time 3)). An ICC(A,3) of .715 (95 % CI [.654, .767]) was observed, indicating good stability across all timepoints.

#### 2.3.2 Biomarkers

##### 2.3.2.1 Hair cortisol concentrations (HCC)

Hair sampling was optional for participants, and samples were collected after obtaining additional informed consent. Hair samples were collected from >80 % of participants in each wave, with *n* = 251 at Time 1, *n* = 201 at Time 2, and *n* = 161 at Time 3. Hair strands in the posterior vertex region of the head were tied off with a thin rubber band. A trained study team member cut these hair strands as close to the scalp as possible. The samples were then enveloped in aluminium foil and stored in a box at room temperature. HCC was analysed in the 1 cm segment of the hair strand closest to the scalp. Assuming an average hair growth of 1 cm/month (Wennig, 2000), this sample represents hair growth over one month prior to sampling.

After each study wave, samples were analysed by Prof. Kirschbaum at the Dresden Lab Service (Dresden, Germany) using a column-switching ionization tandem mass spectrometry assay (LC-ESI-MS/MS). Specifically, hair samples were first rinsed with isopropanol, and steroid hormones were then extracted from 10 mg of whole, non-pulverized hair using methanol incubation. An online solid-phase extraction method employing column switching was used, followed by analyte detection with an AB Sciex ESI 6500+ TripleQuad mass spectrometer. This is an efficient, highly sensitive, and reliable method for quantifying steroid hormones in human hair. The protocol is described in full elsewhere. In contrast to the described methods the Mobile phase A was 100 ml 2 mM NH_4_AC in Methanol, while mobile phase B was 950 ml 2 mM NH_4_AC in methanol. The pH of mobile phase B was adjusted to 4.7 with acetic acid. (Gao et al., 2013; Stalder et al., 2012).All samples were analysed (mean hair mass = 6.6 mg). The intra- and inter-assay coefficients of variation (CVs) for cortisol ranged between 3.7 % and 8.8 % (Gao et al., 2013). An ICC[A,3] of .793 (95 % CI [.748, .831]) was observed, indicating good stability of HCC across the three timepoints.

##### 2.3.2.2 C-reactive protein (CRP)

For analysing high-sensitivity C-reactive protein (CRP), capillary blood samples were collected from all participants. The well-established and minimally invasive dried blood spot method was used, where drops of whole blood from a finger prick are collected on filter papers (McDade et al., 2007). Under sterile conditions, a trained study team member pricked the participant's fingertip with a disposable lancet, and the first drop of blood was wiped away with gauze. Subsequent blood drops of sufficient size were then applied to a filter paper and dried at room temperature for at least 8 h. The samples were stored with desiccant in a sealable multi-barrier pouch at −26 °C. CRP was analysed in the laboratory of the Chair of Health Psychology, Friedrich-Alexander University Erlangen-Nürnberg using a “Human C-Reactive Protein/CRP Quantikine ELISA Kit” (IBL International) (for further details see Becker et al., 2022). The intra-assay CVs were 4.18 % (Time 1), 4.28 % (Time 2), and 4.08 % (Time 3). An ICC[A,3] of .800 (95 % CI [.757, .836]) was observed, indicating good stability of CRP concentrations across the three timepoints.

### 2.4 Statistical analysis

Biological samples that were below detectable limits for the assay were recoded to a score of zero (CRP: 1.01 %, HCC: .9 % of all data). Extreme HCC levels outside 3 SD were trimmed using a winsorising technique (Wilcox, 1994), and CRP values greater than 10 mg/L were excluded as these values likely represent acute inflammatory responses (Pearson et al., 2003).

Missing values were analysed with IBM SPSS Statistics (Version 28), 17.17 % were missing, and Little's MCAR test showed that the data were missing completely at random (χ^2^ = 729.19, *df* = 598, *p* < .001). We imputed data using the following methods: For sex and BMI, missing values at Time 1 were replaced with the within-person means from Time 2 and Time 3; for age, missing values at Time 1 were calculated from within-person values at Time 3 (Time 3 minus 1 year).

For self-report variables, missing individual item response values were replaced with within-person means for that timepoint before calculating the summed scores. For the summed scores of the self-report measures and biosamples, multiple imputation was conducted by creating five imputation data sets and pooling them to replace missing values.

Using Tukey post hoc correction, a series of ANOVA's assessed gender and job position effects for each of perceived stress, wellbeing, CRP and HCC at each timepoint. Then, using the full sample, descriptive analysis, ANCOVAs (covarying for age and sex), and Pearson correlations for associations between key variables were conducted using SPSS. Change scores were calculated for each participant between Time 1 and Time 2, and between Time 2 and Time 3. To examine participants who showed a meaningful change (>1.96 *SD*s in each direction) from Time 1 to Time 2, and from Time 2 to Time 3, Reliable Change Indexes (RCI) were computed for key variables. In the absence of clinical reference norms denoting meaningful change, the z value of 1.96 is used as it constitutes the two-tailed *p <* .05 threshold which suggests change at this level is unlikely due to chance (Blampied, 2022). For PSS4, an RCI of 4.17 was derived using the standard deviation and coefficient alpha from a large, normative data set (Warttig et al., 2013). For the WHO-5 Well-being Index, a clinically relevant change score of 10 points was applied (Topp et al., 2015). For the biosamples, RCIs for HCC and CRP were calculated using the standard deviation and coefficient alpha from the current dataset, yielding RCIs of 9.55 and 1.92, respectively. Participants whose change scores exceeded the respective RCI thresholds were classified as showing a meaningful response (“responders”).

Then, a series of Cross-Lagged Panel Models (CLPM) with Random Intercepts (RI-CLPM) were conducted using MPlus (Version 8.9; Muthén and Muthén, 2017). Traditionally, CLPMs have been used to assess causal effects in longitudinal panel studies by modelling how one variable is associated with changes in another variable at a future timepoint, adjusting for the previous timepoint. Critiques have noted (Hamaker et al., 2015) that these causal estimates are difficult to interpret because they confound between-person stability from within-person change (e.g., Berry and Willoughby, 2017). An RI-CLPM separates the between-person stability (i.e., stable trait-like differences) from within-person (i.e., time variant state-like) changes, allowing for a clearer picture of temporal changes that contribute to evidence in support of causal effects. Consequently, including additional covariates is typically unnecessary when focusing on within-person dynamics, as each individual acts as their own baseline.

All RI-CLPMs were run using maximum likelihood estimation with robust standard errors to account for any skewness in the data (Yuan et al., 2000). We separately estimated RI-CLPMs in which one of the four variables (i.e., PSS4, WHO-5, HCC, CRP) at Time 1 and Time 2 was used to predict the other at Time 2 and 3, respectively. Six series of analyses were conducted, each examining associations between two variables, such that each possible combination of variables (i.e., PSS4 and WHO-5; PSS4 and HCC; PSS4 and CRP; WHO-5 and HCC; WHO-5 and CRP; and CRP and HCC) were examined. Given the focus on within-person changes across time, RI-CLPM analyses overcome the requirement to control for stable demographic variables (e.g., sex), which are accounted for in the random intercept, which adjusts for stable differences across participants.

For example, the first RI-CLPM used the PSS4 score and WHO-5 at Time 1 to predict both variables in the following phases. A random intercept for both PSS4 and WHO-5 was estimated by fixing the factor loadings of each variable at each timepoint to 1 and by allowing two random intercepts to correlate. These random intercepts reflect the sample means of each participant's average perceived stress levels and well-being across all three timepoints. The auto-regressive and cross-lagged within-subjects paths were constrained to equality because we were more interested in the overall effects and temporal ordering of variables, rather than whether these effects changed during the intervals measured. So, for instance, the within-person components of our model estimate a cross-lagged model where the within-person latent variables at Time 3 were regressed onto the within-person latent variables at Time 2, and Time 2 on Time 1.

The covariances between the within-person latent variables at Time 1 were estimated, as well as the contemporaneous residual variables at Times 2 and 3, to account for time-specific sources of systematic variance. The correlations between both random intercepts and the within-person measures of perceived stress and WHO-5 at Time 1 were constrained to 0. Finally, bias-corrected (BC) 95 % confidence intervals (CIs) were estimated using 10,000 bootstrapped resamples with replacements. These CLPM analyses were repeated for all pairs of variables (i.e., PSS4 and HCC; PSS4 and CRP; WHO-5 and HCC; WHO-5 and CRP; CRP and HCC).

Model fit was evaluated using the comparative fit index (CFI), root mean squared error of approximation (RMSEA), and standardised root mean square residual (SRMR). The following cut-offs indicated adequate fit: CFI >.90, RMSEA ≤.06, SRMR ≤.08 (Hu and Bentler, 1999).

## 3 Results

### 3.1 Descriptives

Although not a primary aim, a comparison of sex and job position differences was undertaken at each timepoint for each of perceived stress, wellbeing, HCC, and CRP to assist with future interpretation of the data. At T1, women reported higher stress *F*(1, 294) = 10.20, *p* = .002 and HCC than males, *F*(1, 294) = 4.56, *p* = .033. At T2 there were no sex differences on any of the four variables. At T3, women had higher perceived stress, *F*(1, 294) = 12.00, *p* < .001, lower well-being *F*(1, 294) = 8.65, *p* = .004, and higher HCC *F*(1, 294) = 8.22, *p* = .004 than males. All other comparisons were non-significant (see Supplementary Table 1 for full details). In terms of differences based on job position (carers, medical care, research personnel, medical-technical personnel, administrative personnel and other), medical care staff had lower perceived stress than all other workers (*p* < .05) at T1. At T2, medical care staff had lower CRP concentrations than all other employee groups (*p* < .05). All other comparisons were non-significant (see Supplementary Table 2 for full details).

Using the full sample, descriptive statistics and correlations for perceived stress, well-being, HCC, and CRP are presented in Table 1, Table 2. Pairwise comparisons showed that perceived stress levels increased over time (Table 3). Well-being at Time 2 and 3 was significantly lower than at Time 1, and Well-being at Time 3 was significantly lower than at Time 2. HCC decreased from Time 1 to Time 2 and then increased from Time 2 to Time 3. CRP did not change across timepoints. Perceived stress and well-being were negatively correlated, with elevated perceived stress associated with lower wellbeing scores. HCC at Time 3 was positively associated with well-being at Time 1 and with CRP at all timepoints.

**Table 1:** Demographic characteristics of the study participants.

	*M*	*SD*
Age (years)	28.59	8.83
BMI	23.74	4.79

Gender	*n*	%

Male	66	22.3
Female	230	77.7

Occupation	*n*	%

Carers	84	28.4
Medical care	62	20.9
Research personnel	41	13.9
Medical-technical personnel	40	13.5
Administrative	16	5.4
Other (e.g., midwives, therapists)	50	16.9

**Table 2:** Marginal means (controlling for age and gender) and standard deviations for perceived stress, well-being, hair cortisol concentrations, and low-grade inflammation levels.

Measure	Time 1		Time 2		Time 3		ANCOVAs	
	*M*	*SD*	*M*	*SD*	*M*	*SD*	*F*(*df*); *p*	η^2^
PSS4	6.21	2.79	6.75	2.92	6.92	2.86	10.05(2, 586); <.001	.033
WHO-5	62.53	19.10	56.26	16.80	54.11	17.36	32.93(2, 586); <.001	.100
CRP	1.23	1.74	1.34	1.55	1.36	1.36	1.37(2, 586); .254	.005
HCC	9.94	9.61	5.28	.28	10.29	.47	86.34(2, 586); <.001	.226

*Note.* PSS4 = Perceived Stress Scale. WHO-5 = WHO5 Well-being. CRP = C-reactive Protein Levels (mg/L). HCC = Hair Cortisol Concentrations (pg/mg).

**Table 3:** Correlations between perceived stress, well-being, hair cortisol concentrations, and low-grade inflammation levels across three timepoints.

Measure	Time	PSS4			WHO-5			CRP			HCC		
		Time 1	Time 2	Time 3	Time 1	Time 2	Time 3	Time 1	Time 2	Time 3	Time 1	Time 2	Time 3
PSS4	1	–											
	2	.460∗∗	–										
	3	.461∗∗	.592∗∗	–									
WHO-5	1	−.506∗∗	−.264∗∗	−.245∗∗	–								
	2	−.400∗∗	−.600∗∗	−.459∗∗	−.470∗∗	–							
	3	−.314∗∗	−.475∗∗	−.632∗∗	.323∗∗	.598∗∗	–						
CRP	1	.060	.044	−.016	−.027	.050	.084	–					
	2	.088	.046	−.019	−.046	.030	.045	.603∗∗	–				
	3	.107	.088	.033	−.037	.020	.043	.513∗∗	.626∗∗	–			
HCC	1	.060	.104	.044	.047	−.017	.026	.047	.034	.041	–		
	2	.015	.011	−.019	.077	.076	.073	.111	.092	.059	.582∗∗	–	
	3	.078	.023	−.017	.119∗	.070	.087	.142∗	.195∗∗	.127∗	.643∗∗	.614∗∗	–

*Note. N* = 296. ∗*p* < .05. ∗∗*p* < .001. PSS4 = Perceived Stress Scale 4. WHO-5 = Well-being. CRP = C-reactive Protein Levels (mg/L). HCC = Hair Cortisol Concentrations (pg/mg).

**Table 4:** Proportion of sample showing a reliable change for perceived stress, well-being, low-grade inflammation, and hair cortisol concentrations across time.

	Reliable decrease		Reliable increase		No Reliable Change	
	*n*	%	*n*	%	*n*	%
PSS4 Time 1-Time 2	11	3.7	34	11.4	251	84.5
PSS4 Time 2-Time 3	9	3.0	13	4.4	274	92.3
WHO-5 Time 1-Time 2	114	38.4	41	13.8	141	47.5
WHO-5 Time 2-Time 3	93	31.3	50	16.8	153	51.5
CRP Time 1-Time 2	17	5.7	15	5.1	264	88.9
CRP Time 2-Time 3	13	4.4	12	4.0	271	91.2
HCC Time 1-Time 2	38	12.8	2	.7	256	86.2
HCC Time 2-Time 3	2	.7	41	13.8	253	85.2

*Note. N* = 296. PSS4 = Perceived Stress Scale 4. WHO-5 = Well-being. CRP = C-reactive Protein Levels (mg/L). HCC = Hair Cortisol Concentrations (pg/mg).

Participants showing a meaningful change between Time 1 and Time 2, and between Time 2 and Time 3, were identified. The proportions of participants exhibiting reliable change (“responders”) in perceived stress, well-being, HCC, and CRP are displayed in Table 4.

### 3.2 Temporal associations of perceived stress, well-being, low-grade inflammation, and hair cortisol concentrations

#### 3.2.1 Temporal associations of perceived stress and well-being

The Chi-square (χ^2^) test indicated the data aligned with the theoretical model, χ^2^ (5) = 8.920, *p* = .112. Additionally, fit indices suggested an acceptable model fit: CFI = .994; SRMR = .051; RMSEA = .051 (BC CI_90_ = [.000, .105]).

The autoregressive paths revealed that changes in perceived stress levels from an individual trait level at a preceding timepoint_(T-1)_ were associated with increased deviations at a future timepoint_(T)_ (*B* = .210, BC CI_95_ = [.032, .388], *p* = .021). Also, changes in well-being levels from an individual trait level at a preceding timepoint _(T-1)_ were associated with increased deviations at a future timepoint_(T)_ (*B* = .240, bias corrected (BC) CI_95_ = [.044, .435]; *p* = .016). The random intercepts were significantly negatively associated with each other (*B* = −10.478, BC CI_95_ = [−18.540, −2.416]; *p* = .011), indicating that higher perceived stress levels were associated with lower well-being levels over time between-persons.

**Fig. 1:**
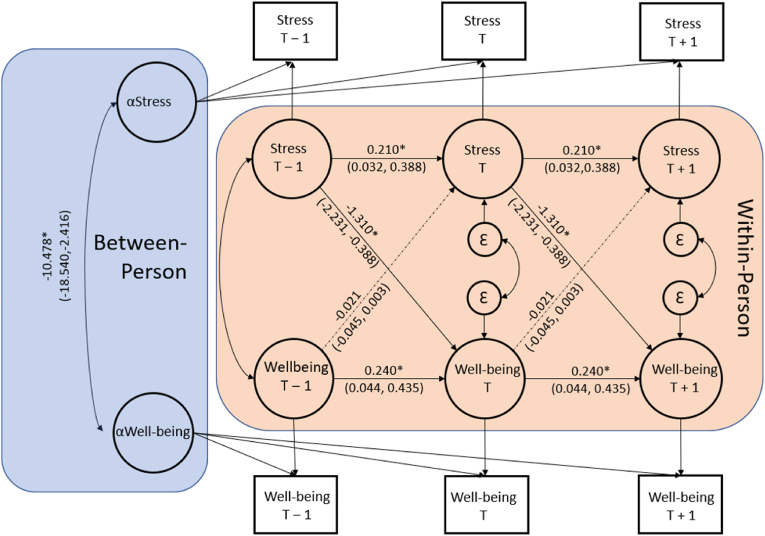
Random Intercepts Cross-lagged Panel Model for Perceived Stress and Well-being *Note.* ∗*p* < .05. Solid lines indicate significant paths. Dashed lines indicate non-significant paths.

Most relevant to our aims were the within-person cross-lagged effects between perceived stress and well-being (Fig. 1). Specifically, increased perceived stress levels from an individual trait level at a preceding timepoint_(T-1)_ were associated with decreases in WHO-5 levels from an individual trait level at a future timepoint_(T)_ (*B* = −1.310, BC CI_95_ = [−2.231, −.388]; *p* = .005), but decreases in well-being levels_(T-1)_ were not associated with increases in perceived stress at a later timepoint (*B* = −.021, BC CI_95_ = [−.045, .003], *p* = .089). This suggests that increases in perceived stress preceded decreases in well-being.

#### 3.2.2 Temporal associations of perceived stress and hair cortisol concentrations

The Chi-square (χ^2^) test indicated that the empirical model supported the theoretical model, χ^2^ (5) = 8.39, *p* = .136. Additionally, fit indices indicated an acceptable model fit with the data: CFI = .992; SRMR = .035; RMSEA = .048 (BC CI_90_ = [.000, .102]).

The autoregressive paths revealed that changes in perceived stress levels from an individual trait level at the preceding timepoint_(T-1)_ were associated with increased deviations at a future timepoint_(T)_ (*B* = .227, CI_95_ = [.030, .424], *p* = .024), but not for changes in HCC (*B* = .098, BC CI_95_ = [−.047, .242], *p* = .185). Furthermore, there were no cross-lagged effects from perceived stress to HCC (*B* = .005, CI_95_ = [−.012, .022], *p* = .552) or from HCC to perceived stress (*B* = .397, CI_95_ = [−.575, 1.369], *p* = .424). This suggested that changes in perceived stress from an individual trait level at a preceding timepoint_(T-1)_ were not associated with changes in HCC from an individual trait level at a future timepoint_(T)_, and vice versa. The random intercepts showed no associations between perceived stress and hair cortisol between-persons across all timepoints (*B* = .041, BC CI_95_ = [−.052, .133], *p* = .387).

#### 3.2.3 Temporal associations of perceived stress and low-grade inflammation

The Chi-square (χ^2^) test indicated that the empirical model significantly deviated from the theoretical model, χ^2^(5) = 11.553, *p* = .042. Nonetheless, fit indices suggested an acceptable model fit: CFI = .988; SRMR = .038; RMSEA = .067, BC CI_90_ [.012, .118].

The autoregressive paths revealed that changes in perceived stress from an individual trait level at a preceding timepoint _(T-1)_ were associated with increased changes at a future timepoint _(T)_ (*B* = .253, CI_95_ = [.068, .439], *p* = .007). Also, changes in CRP levels from an individual trait level at a preceding timepoint_(T-1)_ were associated with increased changes in CRP levels at a future timepoint_(T)_ (*B* = .267, BC CI_95_ = [.097, .437], *p* = .002). However, there were no cross-lagged effects from perceived stress to CRP levels (*B* = .010, BC CI_95_ = [−.013, .033], *p* = .380) or from CRP levels to perceived stress (*B* = −.123, BC CI_95_ = [-.938, .439], *p* = .767). This indicated that changes in perceived stress from an individual trait level at a preceding timepoint_(T-1)_ were not associated with changes in CRP levels from an individual trait level at a future timepoint_(T)_, and vice versa. There were no associations between perceived stress and low-grade inflammation between-persons across all timepoints (*B* = .058, BC CI_95_ = [-.110, .226], *p* = .497).

#### 3.2.4 Temporal associations of well-being and hair cortisol concentrations

The Chi-square (χ^2^) test indicated that the theoretical model was not supported by the empirical data, χ^2^ (5) = 12.655, *p* = .027. More importantly however, the collection of fit indices suggested an acceptable model fit: CFI = .982. SRMR = .050, RMSEA = .072 (BC CI_90_ = [.022, .122]).

The autoregressive paths revealed changes in well-being levels from an individual trait level at a preceding timepoint_(T-1)_ were associated with increased deviations at a future timepoint_(T)_ (*B* = .340, CI_95_ = [.055, .625], *p* = .019), but not for changes in HCC (*B* = .098, CI_95_ = [-.046, .241], *p* = .183). There were no cross-lagged effects from well-being to HCC (*B* = .000, CI_95_ = [-.003, .003], *p* = .977), or from HCC to well-being (*B* = −1.196, CI_95_ = [-7.336, 4.944], *p* = .703). This indicated that changes in well-being from an individual trait level at a preceding timepoint_(T-1)_ were not associated with changes in HCC from an individual trait level at a future timepoint_(T)_ and vice versa. There was also no association between well-being and hair cortisol concentrations between-persons across all timepoints (*B* = .424, CI_95_ = [-.214, 1.061], *p* = .193).

#### 3.2.5 Temporal associations of well-being and low-grade inflammation

The Chi-square (χ^2^) test indicated that the theoretical model was supported by the empirical data, χ^2^ (5) = 10.954, *p* = .052. Additionally, fit indices suggested an acceptable model fit: CFI = .989. SRMR = .047, RMSEA = .063 (BC CI_90_ = [.000, .115]).

The autoregressive paths revealed changes in well-being levels from an individual trait level at a preceding timepoint _(T-1)_ were associated with increased deviations at a future timepoint_(T)_ (*B* = .303, [CI_95_ = .011, .596], *p* = .042), and changes in CRP levels from T-1 were associated with increased changes at T (*B* = .271, BC CI_95_ = [.094, .448], *p* = .003). However, there were no cross-lagged effects from well-being to CRP (*B* = −.002, BC CI_95_ = [-.006, .002], *p* = .359), or from CRP to well-being (*B* = .476, BC CI_95_ = [-5.393, 6.346], *p* = .874). This meant that changes in well-being from an individual trait level at a preceding timepoint_(T-1)_ were not associated with changes in CRP levels from an individual trait level at a future timepoint_(T)_, and vice versa. The random intercept indicated no association between well-being and low-grade inflammation between-persons across all timepoints (*B* = .348, BC CI_95_ = [-1.064, 1.761], *p* = .629).

#### 3.2.6 Temporal associations of low-grade inflammation and hair cortisol concentrations

The Chi-square (χ^2^) test indicated that the model had adequate model fit, χ^2^ (5) = 7.968, *p* = .158. Additionally, fit indices suggested an acceptable fit: CFI: = .994. SRMR = .031, RMSEA = .045 (BC CI_90_ = [.000, .100]).

The autoregressive paths revealed changes in CRP levels from an individual trait level at a preceding timepoint_(T-1)_ were associated with increased deviations at a future timepoint_(T)_ (*B* = .268, BC CI_95_ = [.098, .438], *p* = .002), but not for changes in HCC (*B* = .113, CI_95_ = [−.029, .254], *p* = .119). There was no cross-lagged effect from CRP to HCC (*B* = .095, BC CI_95_ = [−.082, .111], *p* = .210), or from HCC to CRP (*B* = .095, BC CI_95_ = [−.054, .244], *p* = .210). This indicated that changes in CRP levels from an individual trait level at a preceding timepoint_(T-1)_ were not associated with changes in HCC from an individual trait level at a future timepoint_(T)_, and vice versa. The random intercepts suggested no associations between low-grade inflammation and hair cortisol concentrations between-persons across all timepoints (*B* = .002, BC CI_95_ = [−.016, .020], *p* = .847).

## 4 Discussion

### 4.1 Findings

The present study investigated the prospective associations between perceived stress, well-being, HPA-axis function (i.e., HCC), and low-grade inflammation (i.e., CRP). Furthermore, for the first time, we aimed to determine the temporal order of these variables, that is, whether a change in one variable at an earlier point in time preceded changes in another. Our results showed that changes in perceived stress preceded changes in well-being. In examining temporal effects, we found evidence that increases in perceived stress at an earlier time led to decreases in well-being approximately 6 months later; however, changes in well-being leading to changes in perceived stress were non-significant. In our examination of temporal associations between psychological and biological variables, we found no evidence of changes in HCC or low-grade inflammation leading to changes in perceived stress or well-being, nor evidence of changes in stress or well-being leading to changes in hair cortisol concentrations or low-grade inflammation. The autoregressive pathways showed within-variable changes across time for perceived stress, well-being, and low-grade inflammation, but not for HCC, as changes in HCC at an earlier time were not related to changes in HCC at a later time.

Our prospective findings align with several cross-sectional studies that reported no relationship between perceived stress and HCC (Dowlati et al., 2010; O'Brien et al., 2013; Skoluda et al., 2012; Wells et al., 2014), though others found negative relationships (Karlén et al., 2011) or positive associations (Faresjö et al., 2014; Kalra et al., 2007). Similarly, prospective examinations have yielded mixed findings. Like our study, some studies reported no prospective stress-HCC relationships (Gidlow et al., 2016; Holding et al., 2021; Janssens et al., 2017; Kramer et al., 2009; Mayer et al., 2018; Planert et al., 2023). However, others have found negative relationships based on changes over time (Penz et al., 2019; Stalder et al., 2012).

Stalder's meta-analysis (2017) found no association between perceived stress and HCC. Stalder's findings are at odds with other meta-analyses that examined stress and other cortisol measures. Specifically, previous meta-analyses examining the relationship between cortisol awakening response and stress demonstrated a positive association with workplace stress (Chida and Steptoe, 2009; Eddy et al., 2023) and general stress (Chida and Steptoe, 2009). While these meta-analyses found positive relationships, the effect sizes were small. Furthermore, analysis of cortisol in blood, saliva, and urine is subject to differences in diurnal circadian rhythms, pulsatile bursts (Lightman et al., 2008) and acute stress (Hellhammer and Schubert, 2012). These measures are indicative of short-term levels of circulating cortisol and stress reactivity, whereas HCC provides valuable information about the effects of chronic stress and prolonged cortisol secretions.

To address the limitations of traditional cortisol sampling measures that capture brief fluctuations, our study utilised HCC as a stable, reliable biomarker of long-term HPA-axis activity. While advances in biosampling through the estimation of HCC provide advantages over fluid-based cortisol measures, it is not without concerns. Staufenbiel et al. (2013) noted that many of the studies collected hair sample lengths that represent an average of three months of accumulative stress, but the PSS4 measures the preceding one-month period; they suggest there may be a time lag between the two measurements. Our study addresses this by collecting hair samples that represent one month of growth. As the methodological advantages of measuring cortisol accumulation in hair samples may provide a more sophisticated approach, the lack of association seen may suggest the effect of chronic stress on accumulated cortisol is more tenuous than previously thought and other pathways between perceived stress and health should be considered (Miller et al., 2007). For example, specific types of stress (work, home, etc.) may be more associated with cortisol than others. For instance, a subset of the data from this paper showed that general workplace stress was not prospectively associated with HCC; however, technostress was negatively associated with HCC (Kaltenegger et al., 2024).

Unlike an earlier systematic review (Johnson et al., 2013) of 40 articles investigating chronic psychosocial stress and CRP, we found no association between perceived stress and CRP. In a large Whitehall II study (*N* = 2528), workplace stress was not associated with CRP but was associated with IL-6 in women (Piantella et al., 2021b). Prospective investigations of workplace stress and inflammatory markers provided inconclusive results (Magnusson Hanson et al., 2019; Shirom et al., 2008). For example, Magnusson Hanson et al. (2019) found a lagged association between social support and IL-6, but not between job demands or job control and IL-6. However, they found prospective associations between job demands and IL-6, as well as cross-sectional associations between job demands and CRP, and job strain and CRP (Magnusson Hanson et al., 2019). The mixed results may suggest a complex interplay between aspects of workplace stress and measures of immunity. Cuitún Coronado et al. (2018) demonstrated a relationship between the cumulative effects of workplace stress, as measured by an ERI imbalance, and an allostatic load index of measures across physiological systems associated with stress. Our study assessed low-grade inflammation with only CRP; future stress research should consider additional biomarkers of inflammation (e.g., IL-6, IL-10, TNF/IL-10).

In addition to the primary objective of determining the temporal associations between variables, our study examined the reliable change for each measure across time. Our results show that for perceived stress, HCC, and low-grade inflammation, the large majority of participants (<84.5 %) showed no meaningful change across time. This may in part explain the lack of prospective changes between variables. However, close to half of the participants showed a meaningful change in well-being across time, with most of these showing a decrease in well-being the longer they were employed in the position. The mean PSS4 scores fell within the normal range (*M* = 6.11, *SD* = 3.14; Warttig et al., 2013), and this may explain why only some participants showed a reliable change. These findings may also suggest that the PSS4, along with biological measures of HCC and CRP, are less sensitive to temporal changes than the WHO-5.

Our findings indicate that elevated perceived stress precedes declines in well-being, and not the reverse. Reduced well-being has been linked to increased risk of depression, with the WHO-5 serving as a valid screening tool for depressive symptoms (Topp et al., 2015). Preventing declines in well-being is clinically important, as depression is the most prevalent psychological disorder globally, affecting both physical and mental health and the leading contributor to disability worldwide (Liu et al., 2020). These results suggest that early interventions targeting perceived stress may help buffer against subsequent reduced well-being. Identifying individuals who show early signs of stress, such as the small subset of our sample with significant elevations, may allow employers to target intervention and prevention efforts on those most at risk.

### 4.2 Limitations

Our findings should be considered in light of some limitations. Selection bias may have occurred such that participants with particularly high stress may have been hesitant to participate in the study, as it may have added a further burden. However, as participants were recently employed staff who had undergone medical checks, this may be representative of a healthy workforce. Nevertheless, the reported changes in stress levels were sufficient to lead to changes in well-being. The generalisability of our findings may be constrained, as the participants were recruited solely in a single healthcare setting. The generalisability may be further limited by our sample being predominantly young females; however, this is representative of the healthcare workforce. Although we assessed gender differences on the key variables (Supplementary Table 1), the effects of gender were not analysed in this study of lagged relationships between variables. Our prospective design, however, provided the minimum of three measurement waves required to control for stable between-person differences in RI-CLPM Hamaker et al. (2015). Finally, the Perceived Stress Scale (Cohen et al., 1983), while well-supported in the literature for its psychometric properties, does not necessarily assist in identifying a targeted intervention, as the measure does not specify the specific source of stress (work, home, caregiver, etc.). Additionally, the sensitivity of the PSS-4 to detect within person changes in low stress employees has been questioned recently (Cook et al., 2024), and our findings align with these results with approximately 50 % showing reliable changes on the WHO-5 compared to only 10 % on the PSS-4. Replication of our findings in older professionals, samples with a higher proportion of males, as well as outside of healthcare is still needed.

### 4.3 Conclusion

Our prospective cohort study did not support a directional association whereby changes in stress predicted subsequent changes in either HCC or low-grade inflammation. These findings challenge the predictive use of single biological markers in measuring the physiological aspects of psychological stress, and they highlight the need for more sophisticated biomarker approaches that use combinatorial measures or ratios of pro-inflammatory to anti-inflammatory ratios (e.g., Piantella et al., 2022) and research designs. Longitudinal studies using combinatorial biological markers may assist in clarifying the mechanisms by which psychological stress contributes to physiological dysregulation, health deterioration and disease.

## CRediT authorship contribution statement

**Monica T. Jones:** Writing – review & editing, Writing – original draft, Investigation, Formal analysis, Data curation. **Rachael A. Cronin:** Writing – review & editing, Supervision, Investigation, Data curation. **Mathew D. Marques:** Writing – review & editing, Writing – original draft, Validation, Methodology, Formal analysis, Data curation. **Matthias Weigl:** Writing – review & editing, Resources, Project administration, Methodology, Investigation, Data curation, Conceptualization. **Nicolas Rohleder:** Writing – review & editing, Resources, Project administration, Methodology, Investigation, Funding acquisition, Conceptualization. **Linda Becker:** Writing – review & editing, Resources, Project administration, Methodology, Investigation, Funding acquisition, Conceptualization. **Helena C. Kaltenegger:** Writing – review & editing, Writing – original draft, Resources, Project administration, Methodology, Investigation, Formal analysis, Data curation, Conceptualization. **Bradley J. Wright:** Writing – review & editing, Writing – original draft, Supervision, Investigation, Formal analysis.

## Funding statement

This work is part of the research project “*Identifikation biomedizinischer und gesundheitlicher Wirkweisen von positiven und negativen Auswirkungen von digitalem Stress und dessen Bewältigung”* [‘Identification of biomedical and health-related modes of action of positive and negative effects of digital stress and coping with it’] which is part of the Bavarian Research Association on Healthy Use of Digital Technologies and Media (ForDigitHealth), funded by the Bavarian Ministry of Science and Arts. HCK, MW and DN have been partly funded by the Munich Centre for Health Sciences (MC-Health). The funders were not involved in the study design, the collection, analysis and interpretation of the data, the writing of the report, and the decision to submit the paper for publication.

The authors have no competing interests to declare.

## Declaration of competing interest

The authors declare that they have no known competing financial interests or personal relationships that could have appeared to influence the work reported in this paper.

## Data Availability

Data will be made available on request.
