# Supplementary material for: Perceived stress precedes declines in Well-being: A prospective study of stress, well-being, hair cortisol, and low-grade inflammation in hospital employees
Source: Brain Behav Immun Health. 2025 Dec 11;51:101158. doi: 10.1016/j.bbih.2025.101158 (PMC12775997; doi:10.1016/j.bbih.2025.101158)
Supplement: Multimedia component 1 [file mmc1.docx]

Supplementary Table 1. Comparison of Gender Differences for each of PSS4, WHO-5, CRP, and HCC at Timepoints 1-3.

|  |  |  | Male | | Female | | ANOVAs |  |  |
| --- | --- | --- | --- | --- | --- | --- | --- | --- | --- |
|  |  | Measure | *M* | *SD* | *M* | *SD* | *F*(*df*); *p* | η^2^ |  |
| Time 1 |  | PSS4 | 5.26 | 2.80 | 6.48 | 2.73 | 10.20(1, 294); .002 | .002 |  |
|  |  | WHO-5 | 64.06 | 19.64 | 62.09 | 18.96 | 0.55(1, 294); .460 | .460 |  |
|  |  | CRP | 1.13 | 1.52 | 1.25 | 1.81 | 0.28(1, 294); .600 | .600 |  |
|  |  | HCC | 7.73 | 5.87 | 10.58 | 10.36 | 4.56(1, 294); .033 | .033 |  |
| Time 2 | |  | PSS4 | 6.15 | 2.80 | 6.92 | 2.94 | 3.59(1, 294); .059 | .012 |
|  | |  | WHO-5 | 58.55 | 16.70 | 55.60 | 16.80 | 1.58(1, 294); .210 | .005 |
|  | |  | CRP | 1.21 | 1.42 | 1.38 | 1.58 | 0.56(1, 294); .449 | .002 |
|  | |  | HCC | 5.17 | 5.19 | 5.31 | 4.89 | 0.04(1, 294); .837 | .000 |
| Time 3 | |  | PSS4 | 5.86 | 2.95 | 7.22 | 2.77 | 12.00(1,294); < .001 | .039 |
|  | |  | WHO-5 | 59.58 | 18.59 | 52.54 | 16.70 | 8.65(1, 294); .004 | .029 |
|  | |  | CRP | 1.18 | 1.02 | 1.41 | 1.44 | 1.55(1, 294); .213 | .005 |
|  | |  | HCC | 10.29 | 8.80 | 11.01 | 8.16 | 8.22(1, 294); .004 | .027 |

Supplementary Table 2. Comparison of Occupational Position Differences for each of PSS4, WHO-5, CRP, and HCC at Timepoints 1-3.

|  |  | Carers | | Medical Care | | Academics | | Medical-technical | | Administrative | | Other | | Significant  Difference |  |
| --- | --- | --- | --- | --- | --- | --- | --- | --- | --- | --- | --- | --- | --- | --- | --- |
|  | Measure | *M* | *SD* | *M* | *SD* | *M* | *SD* | *M* | *SD* | *M* | *SD* | *M* | *SD* |  |  |
| Time 1 | PSS4 | 6.45 | 2.42 | **5.05*** | 3.14 | 6.05 | 2.47 | 7.38 | 2.83 | 6.25 | 2.59 | 6.24 | 2.73 | Yes |  |
|  | WHO-5 | 61.52 | 18.86 | 68.52 | 19.31 | 63.32 | 14.53 | 57.70 | 21.09 | 58.50 | 12.47 | 60.88 | 21.42 | No |  |
|  | CRP | 1.16 | 1.59 | 1.07 | 1.49 | 1.48 | 1.98 | 1.86 | 2.68 | 1.09 | 1.08 | 0.93 | 1.20 | No |  |
|  | HCC | 11.34 | 10.57 | 8.95 | 7.52 | 8.85 | 5.58 | 9.73 | 10.50 | 11.63 | 14.09 | 9.64 | 10.70 | No |  |
| Time 2 | | PSS4 | 7.55 | 2.50 | 5.87 | 3.01 | 6.49 | 2.94 | 7.25 | 2.94 | 6.06 | 3.19 | 6.48 | 3.09 | No |
|  | | WHO-5 | 53.90 | 15.37 | 60.00 | 16.81 | 57.85 | 16.69 | 55.30 | 17.98 | 56.25 | 12.94 | 54.72 | 19.33 | No |
|  | | CRP | 1.41 | 1.55 | **0.93*** | 0.96 | 1.52 | 1.84 | 1.97 | 2.09 | 1.10 | 0.93 | 1.19 | 1.44 | Yes |
|  | | HCC | 5.77 | 4.29 | 4.77 | 5.20 | 4.19 | 3.13 | 5.15 | 3.58 | 6.60 | 9.77 | 5.80 | 5.94 | No |
| Time 3 | | PSS4 | 7.37 | 2.52 | 5.97 | 3.18 | 6.73 | 2.90 | 7.43 | 2.52 | 7.69 | 3.66 | 6.80 | 2.56 | No |
|  | | WHO-5 | 53.48 | 17.15 | 59.03 | 17.15 | 51.90 | 15.91 | 52.10 | 17.51 | 52.75 | 20.41 | 53.28 | 18.15 | No |
|  | | CRP | 1.52 | 1.45 | 1.18 | 1.32 | 1.26 | 1.29 | 1.74 | 1.33 | 1.26 | 1.29 | 1.20 | 1.44 | No |
|  | | HCC | 11.43 | 7.88 | 8.53 | 4.39 | 9.61 | 7.03 | 9.19 | 5.45 | 11.56 | 13.73 | 11.91 | 11.72 | No |

Note. Significant differences based on Tukey post hoc comparison test where *p* < .05. * denotes that medical care workers are lower than all other workers for PSS4 at Time 1 and CRP at Time 2
